# Interventional treatment of post tracheostomy tracheal stenosis in neurological rehabilitation: results of a single-center registry

**DOI:** 10.3389/fresc.2026.1776925

**Published:** 2026-03-12

**Authors:** Lukas Ley, Pascal Klingenberger, Tamara Schlitter, Jürgen Hetzel, Martin Groß, Jens Allendörfer, Dirk Bandorski

**Affiliations:** 1 Campus Kerckhoff, Justus-Liebig-University Giessen, Bad Nauheim, Germany; 2 Neurological Clinic Bad Salzhausen, Nidda, Germany; 3 Department of Pneumology, University Hospital Basel, Basel, Switzerland; 4 Faculty of Medicine, University of Tübingen, Tübingen, Germany; 5 Deutsche Interdisziplinäre Gesellschaft für Außerklinische Beatmung und Intensivversorgung (DIGAB) e.V., Freiburg, Germany; 6 MEDIAN Klinik Bad Tennstedt, Bad Tennstedt, Germany; 7 Department of Health Services Research, Faculty of Medicine and Health Sciences, Carl von Ossietzky Universität Oldenburg, Oldenburg, Germany; 8 Faculty of Medicine, Semmelweis University Campus Hamburg, Hamburg, Germany

**Keywords:** cryoablation, intervention, outcome, post tracheostomy tracheal stenosis, registry, therapy, tracheal stenosis, treatment

## Abstract

**Introduction:**

Post tracheostomy tracheal stenosis (PTTS) is a serious long-term complication of tracheostomies. PTTS can significantly delay respiratory weaning, impair the patient's quality of life and cause significant healthcare costs. There do not exist any standardized treatment recommendations for PTTS. The aim of the present study was to improve knowledge regarding PTTS treatment and the associated clinical course.

**Methods:**

Consecutive patients who were admitted in an intensive care unit (ICU) of a German neurological specialist hospital from 30.04.2020 to 10.11.2025 were included in a single-center, combined retro- and prospective registry.

**Results:**

132 patients (50.8% female, mean age: 67 years) underwent a total of 198 interventions (mean: 1.5) for the treatment of PTTS. Most patients (68.2%) were treated only once. Cryoablation was the most performed intervention. 70.5% of patients were treated with prednisolone, which was sufficient alone in 30.1% of these patients. 77.3% of patients could be decannulated after PTTS treatment, 17.4% were discharged with a permanent tracheostoma mainly due to their severe neurological condition and 4.6% died during the hospital stay. Mean follow-up was 68 days. Only 2.3% of patients underwent surgical PTTS treatment and 0.8% were in need for an airway stent. All interventions were performed without procedure-associated complications.

**Conclusion:**

Interventional treatment of PTTS in neurological rehabilitation appears to be effective in enabling decannulation, and safe.

## 1 Introduction

The first references to tracheotomy procedures date back to ancient Egypt more than five thousand years ago (1). Tracheostomy techniques have been significantly refined to date and tracheostomies are now widely performed as percutaneous dilatational tracheostomy (PDT) as standard of care in intensive care medicine because they are advantageous for long-term ventilation and subsequent weaning (2–5). However, there exists a serious long-term complication of tracheostomies that can significantly delay respiratory weaning, impair the patient's quality of life and cause significant healthcare costs: post-tracheostomy tracheal stenosis (PTTS) (2, 6, 7). Due to scarce data, the exact incidence is unknown. However, it is assumed that mild, asymptomatic forms occur in almost all patients undergoing tracheostomy, while severe, symptomatic forms are described in only a minority of patients (about 1%–2%) after tracheostomy (8–11). Associated clinical manifestations, including dyspnea, wheezing, coughing, sputum retention, recurrent respiratory infections, stridor, failure of respiratory weaning, and unsuccessful decannulation, may substantially compromise the weaning process and adversely affect the patients' quality of life, thereby underscoring the necessity for comprehensive and individualized therapeutic strategies (12–15). However, there is no standardized treatment strategy for PTTS to date. In general, an interventional procedure is initially suggested, while surgery is recommended for refractory, usually complex cases (13, 16, 17). Moreover, there is no consensus regarding the preferred interventional treatment method for PTTS. Since data in this field are scarce, this registry study reports on the outcomes following interventional treatment of neurological patients with PTTS who were treated with a variety of interventional modalities. The aim of the present paper is to improve knowledge regarding the optimal treatment strategy for patients with PTTS. The aim of this registry study was to conduct a large, structured, and systematic data collection and subsequent analysis on the treatment and clinical course of patients with PTTS. On this basis, the present data may contribute to improving the treatment of PTTS patients.

## 2 Method

### 2.1 Study design

The present study was conducted as a single-center, combined retro- and prospective registry study in the intensive care unit (ICU) of a German neurological specialist hospital. The registry is ongoing prospective since February 2024. Consecutive patients who were admitted from the 30.04.2020 to 10.11.2025 (discharge of the last patient included in the present analysis) were reviewed regarding the in- and exclusion criteria, which have been described in a preceding study (16).

Inclusion criteria:

- Confirmed PTTS by tracheoscopy with exophytic and intraluminal located granulation tissue based on a visual evaluation at the discretion of the endoscopist

Exclusion criteria:

- Laryngeal involvement in the stenosis
- Unstable trachea, e.g., with several cartilage fractures
- <18 years of age
- Pregnant women, breastfeeding mothers and women of childbearing age who refused a pregnancy test on admission
- Lack of consent to the study

A positive ethics vote of the Ethics Committee of the Medical Association of Hesse (Landesärztekammer Hessen) dated 30.05.2023 is available (2022-3199-evBO). The study was officially registered (NCT05924087).

### 2.2 Tracheostomy

All on-site tracheostomies (Tracoe Experc Tracheostomy Kit, TRACOE medical GmbH, Nieder-Olm, Germany) were performed as either direct or indirect PDTs (4, 5). The following process has also been described before (16, 18): Briefly, the puncture site was chosen based on anatomical landmarks. After administration of local anesthesia, the endotracheal tube was retracted, and the puncture site was marked using diaphanoscopy. Direct PDTs were performed as follows: After puncturing the trachea between the 1st and 2nd, or the 2nd and 3rd tracheal ring under aspiration and bronchoscopic control, a skin incision and targeted preparation along the guidewire was performed. After insertion of a conical dilator, the tracheal cannula was placed. Indirect PDTs were performed as follows: After the skin incision and blunt dissection, the trachea was punctured between the 1st and 2nd, or the 2nd and 3rd tracheal ring under aspiration and bronchoscopic control. After insertion of a conical dilator, the tracheal cannula was placed. The endotracheal tube was then completely removed, and the tracheostomy tube fixed and connected to the ventilator.

### 2.3 Tracheoscopy

The suspicion of PTTS arose if symptoms such as dyspnea, severe respiratory effort, stridor or drop in oxygen saturation occurred during the attempt of decannulation. Moreover, every patient was evaluated by an occupational therapist and, if necessary, a linguist because they are mostly in charge of the clinical handling of tracheal cannulas. To confirm symptomatic PTTS and to plan interventional treatment, a tracheoscopy (Olympus BF-1TH1100 EVIS X1 Video Bronchoscope, Olympus, Tokyo, Japan) was performed. After temporarily removing the tracheal cannula, the morphology and extent of the stenosis were tracheoscopically analyzed and the most suitable endoscopic treatment modality was selected and applied. About ten days after each interventional procedure a follow-up tracheoscopy was carried out to review the therapeutic effect. Patients were decannulated if previous symptoms (e.g., dyspnea, severe respiratory effort, stridor, or drop in oxygen saturation during the decannulation) had disappeared, the patient was eupnoeic, had a stable oxygen saturation, was able to cough sufficiently, there was no aspiration risk, and PTTS was visually <50%, mostly <30%, at the discretion of the treating physician. Asymptomatic patients without signs and symptoms of PTTS, e.g., patients who could easily be decannulated, did not undergo tracheoscopy.

### 2.4 Interventional methods

**Figure 1:**
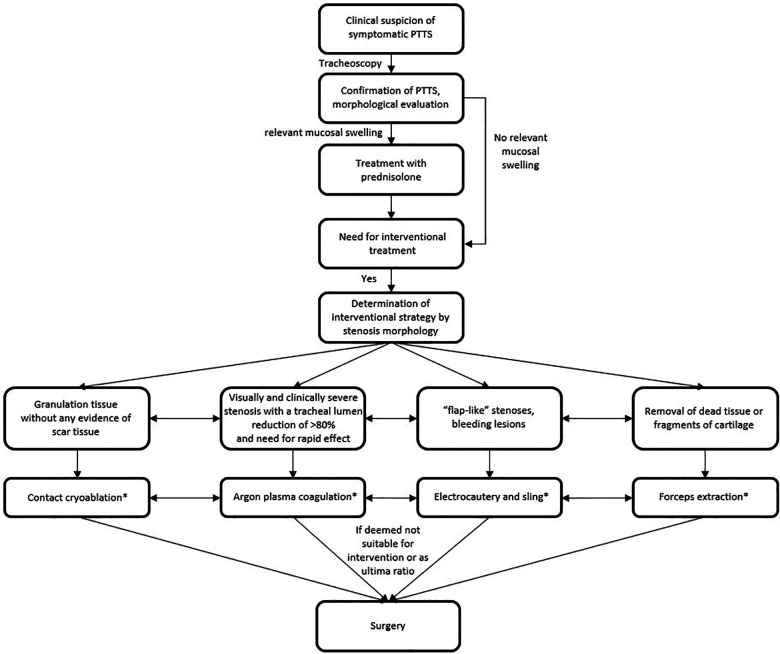
Treatment flowchart. Legend: *: combinations of interventional methods were applied for stenoses with several different morphologies not suitable for only one single modality and depending on the results of a clinical evaluation and the tracheoscopic follow-up, interventions had to be repeated due to remaining or reappearing granulation tissue and persistent symptoms; PTTS, post tracheostomy tracheal stenosis.

Depending on the extent and morphology of the stenosis as well as the severity of symptoms, various interventional treatment methods were used (Figure 1). All eligible patients (PTTS caused by granulation tissue, no scar tissue) were primarily treated with contact cryoablation (KRYO 2, ERBE, Germany). Otherwise, other methods such as argon plasma coagulation (APC; APC 2, ERBE, Germany; for patients with visually and clinically severe stenosis with a tracheal lumen reduction of >80% and need for rapid effect), electrocautery (MTW, Wesel, Germany; e.g., “flap-like” stenoses, bleeding lesions), forceps extraction (removal of dead tissue or fragments of cartilage) or surgery (ultima ratio) were used. Combinations of interventional methods were applied for stenoses with several different morphologies not suitable for only one single modality, e.g., broad-based elevated granulation tissue suitable for cryoablation and “flap-like” tissue non-suitable for cryoablation but electrocautery. Moreover, in case of mucosal swelling patients were treated with prednisolone. Prednisolone was applied directly after PTTS diagnosis for five days each with a daily dose of 40 mg without tapering. Treatment success was controlled after 10 days. Additional prednisolone treatment was then individually applied depending on the results of the control tracheoscopy, e.g., in case of residual swelling at the treating physician discretion. If continued for more than five days, prednisolone was tapered every few days (40 mg, 30 mg, 20 mg, 10 mg, 5 mg, 2.5 mg). Depending on the results of a clinical evaluation and the tracheoscopic follow-up, interventions had to be repeated due to remaining or reappearing granulation tissue and persistent symptoms. All interventions were made with a flexible tracheoscope (Olympus BF-1TH1100 EVIS X1 Video Bronchoscope, Olympus, Tokyo, Japan) and a spiral tube. Anesthesia was achieved with midazolam and propofol. Periprocedural monitoring included 3-lead ECG, oxygen saturation, respiratory rate and non-invasive blood pressure. After the procedure patients were observed on an intermediate care (IMC) ward. Energy settings as well as number and duration of application were individually selected by the treating physician depended on lesion volume and morphology. For contact cryoablation, this has been described before (16).

### 2.5 Statistics

A statistical analysis was performed using Jamovi (Version 2.7, Sydney, Australia). Categorial variables are presented as numbers and percentages. Continuous variables are presented as mean and standard deviation (SD) or median and interquartile ranges (IQR), as appropriate. The chi-square test was used to test the relation between variables. To prevent alpha error accumulation, the *p*-value was adjusted using Bonferroni correction (0.05/9). A *p*-value of <0.006 was therefore considered statistically significant.

## 3 Results

Since the start of the registry in April 2020, 1,450 patients with tracheostomies (including 1,000 referrals) have been treated in our neurological ICU and 450 tracheostomies were performed on site. The most common underlying conditions that led to treatment in the ICU were ischemic and hemorrhagic strokes and critical illness polyneuropathy and myopathy. To date, 132 patients (50.8% female, mean age: 67 ± 13 years) have been treated for PTTS (Supplementary Figure S1). This corresponds to an incidence of symptomatic PTTS that initially impeded decannulation and respiratory weaning of 9.1%. 93.9% of tracheotomies were primary tracheostomies, 74.2% of tracheostomies had already been performed externally, and 9.9% of tracheostomies were performed surgically (Table 1). The underlying conditions that led to treatment in the ICU are shown in Supplementary Table S1.

**Table 1:** Tracheostomy data of patients with post tracheostomy tracheal stenosis.

Parameter	Total	On-site	Referrals
Total, *n* (%)	132 (100)	34 (25.8)	98 (74.2)
First tracheostomy, *n* (%)	124 (93.9)	30 (88.2)	94 (95.9)
Re-tracheostomy, *n* (%)	8 (6.1)	4 (11.8)	4 (4.1)
Percutaneous dilatational tracheostomy, *n* (%)	119 (90.2)	32 (94.1)	87 (88.8)
Surgical tracheostomy, *n* (%)	13 (9.8)	2 (5.9)	11 (11.2)

Data on potential tracheal cartilage fractures after tracheostomy were available for 82 patients. Of these, 29 patients had a tracheal cartilage fracture (22.0% of all PTTS patients, 35.4% of PTTS patients with available data). Tracheal cartilage fractures were significantly more common in women (69.0%) than in men (31.0%, *p* < 0.001). The mean time between tracheostomy and diagnosis of PTTS was 61 ± 34 days. Indications for interventional treatment of PTTS were clinical symptoms (dyspnea, stridor, drop of oxygen saturation) during the process of tracheostomy tube removal in every patient. All PTTSs were simple, located directly above the tracheostoma and visually over 50%. Lesion length was mostly <5 mm but never >10 mm. No patient suffered from multiple granulations.

**Figure 2:**
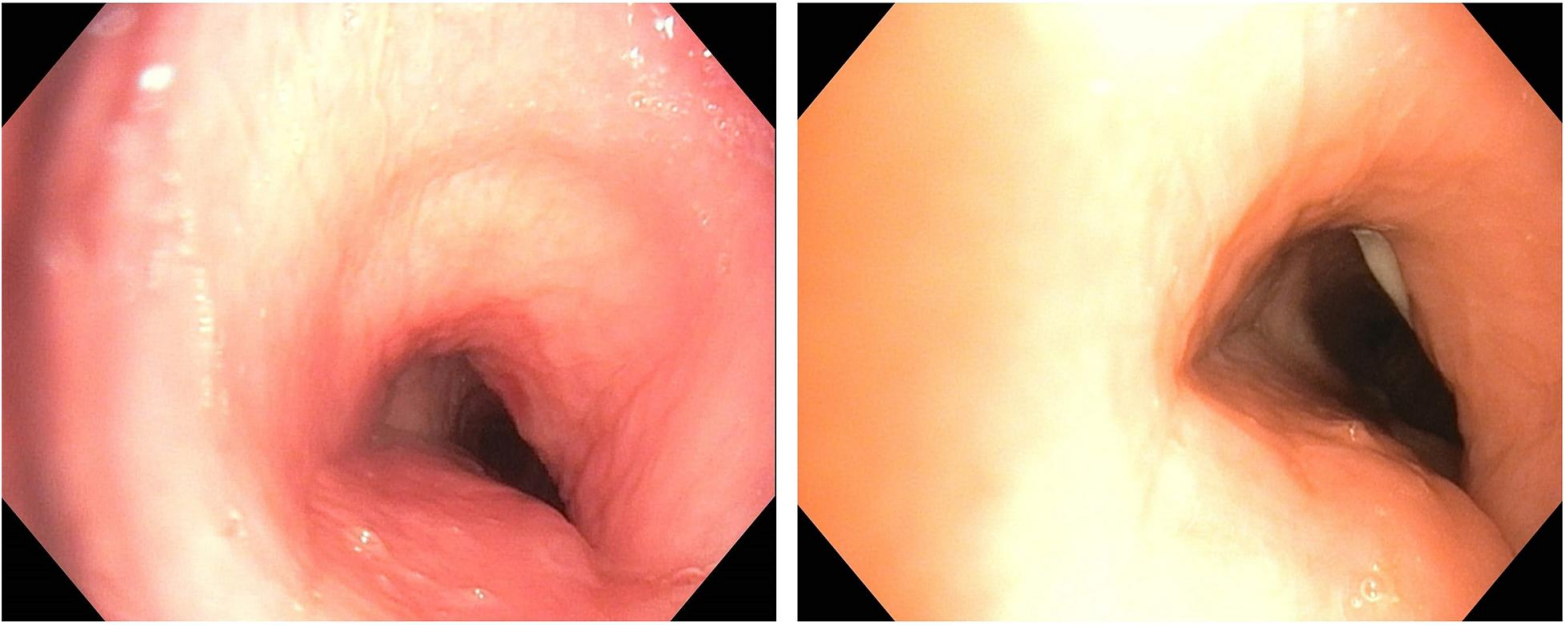
Patient example: prednisolone treatment. Legend: left: before prednisolone, right: after prednisolone, significant reduction in swelling and redness of the mucosa with widening of the tracheal lumen.

**Figure 3:**
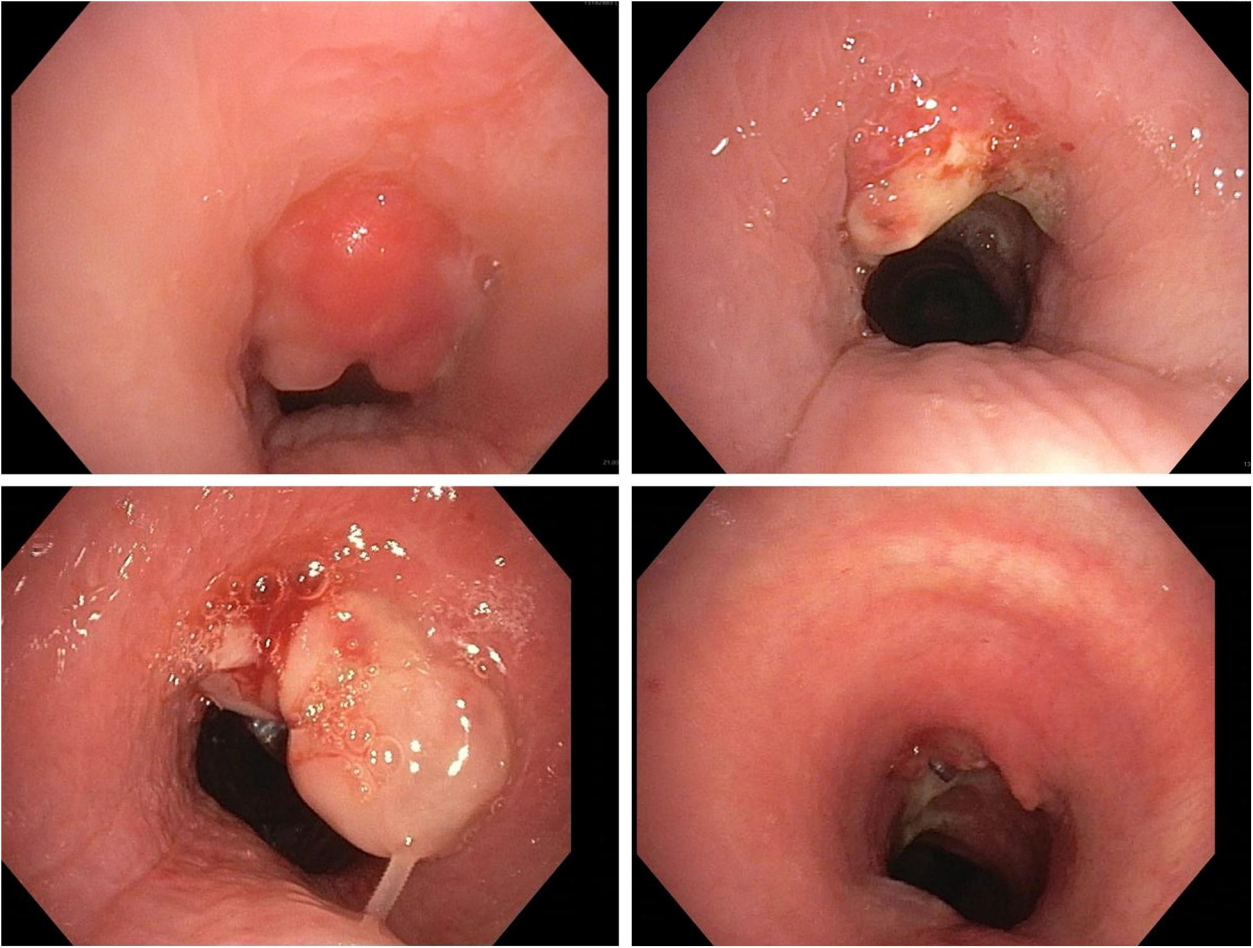
Patient examples: cryoablation and sling treatment. Legend: first patient: top left: before cryoablation, top right: after cryoablation; second patient: bottom left: before sling, bottom right: after sling; results: significant widening of the tracheal lumen.

A total of 198 interventional procedures were performed on 132 patients (mean: 1.5 ± 0.9) to treat PTTS (Figures 2, 3). Of the 198 interventions, cryoablation was performed 117 times (59.1%), sling 56 times (28.3%), APC 10 times (5.1%), external therapy (treatment already performed in the referring hospitals) 4 times (2.%) and forceps 7 times (3.5%). Three patients (2.3%) needed surgical treatment and one patient (0.5%) received an airway stent. All interventions were performed without procedure-associated complications. Most patients (68.2%) were treated only once, while 19.0%, 8.3%, 3.8%, and 0.8% required two, three, four, and five sessions, respectively. Time to re-intervention was 7–10 days in each patient. There was no significant relation between the number of total interventions per patient and the tracheostomy technique (PDT vs. surgical tracheostomy, *p* = 0.891, Supplementary Table S2), number of tracheostomies (primary vs. re-tracheostomy, *p* = 0.640, Supplementary Table S3), presence of tracheal cartilage fracture (*p* = 0.277, Supplementary Table S4), type of initial intervention (*p* = 0.833, Supplementary Table S5) and outcome (*p* = 0.162, Supplementary Table S6).

70.5% of patients were treated with oral or intravenous prednisolone. Of these, 30.1% of patients (*n* = 28/93) were treated with prednisolone alone, and 69.9% of patients (*n* = 65/93) were additionally treated with an interventional modality (86.2% cryotherapy, 6.2% sling, 3.1% external therapy, 1.5% APC, 1.5% surgery, 1.5% forceps). Patients treated with prednisolone alone had significantly more mucosal swelling, but apart from this, there was no significant difference in stenosis morphology or outcome (Supplementary Table S1^1^). Besides temporary blood glucose elevations, there were no adverse events potentially caused by prednisolone. After a mean follow up of 66 ± 33 days, 77.3% of patients could be decannulated after PTTS treatment, 17.4% were discharged with a permanent tracheostoma and 4.6% died during the hospital stay (Supplementary Table S7). Total time to decannulation was 10–50 days depending on the total number of interventions. In the decannulated patients there was no symptomatic PTTS recurrence. Outcome was not statistically significantly different by tracheostomy technique (PDT vs. surgical tracheostomy, *p* = 0.827, Supplementary Table S7), number of tracheostomies (primary vs. re-tracheostomy, *p* = 0.621, Supplementary Table S8), presence of tracheal cartilage fracture (*p* = 0.277, Supplementary Table S9), number of interventions (*p* = 0.162, Supplementary Table S10), and type of initial intervention (*p* = 0.833, Supplementary Table S11).

## 4 Discussion

The present findings show that interventional treatment of PTTS was safe (procedure-associated complication rate: 0%) and effective (decannulation rate: 77.3%) despite symptomatic PTTS that initially impeded decannulation and respiratory weaning. A total of 198 interventions were performed (mean: 1.5) to treat PTTS. Most patients (68.2%) only needed to be treated once. In most of the interventions (59.1%) cryoablation was applied. One of the patients (0.8%) was in need for an airway stent and only three patients (2.3%) underwent surgical PTTS treatment.

In previous studies evaluating the effectiveness of various interventional modalities to treat benign tracheal stenosis [mostly post intubation tracheal stenosis (PITS) and/or PTTS], interventions were also performed repeatedly (mean interventions per patient: 0.9–3.4). Moreover, successful decannulation rates varied from 47%–71% after non-surgical interventions and when including studies with surgical treatment up to 85% (6, 11, 13, 15, 16, 19–27). Therefore, our results are well in line with current literature. However, the present decannulation rate is slightly higher than in previous studies, which may be due to the high number of patients treated in our center, mostly by one operator, and the therefore richer experience in interventional treatment of PTTS. This is underpinned by a slightly higher decannulation rate (77.3%) than in an earlier report from our registry (70%), which suggest a relevant learning curve (16). However, the remaining 22.7% who could not be decannulated should not be judged as treatment failures *per se*, as the main cause of persistent inability to decannulate patients were distinct factors associated to neurological impairment (“neurologically limited”, 78.3%, *n* = 18/23), e.g., neurological severity, dysphagia, absent protective reflexes, aspiration risk or secretion burden. Thus, true weaning failure (“airway limited”) with permanent tracheostoma was only present in 3.8% (21,7%, *n* = 5/132) of all patients, that could not be decannulated because granulations tissue could not be controlled or further interventions were declined by the patient.

In the present study 198 interventional procedures were performed on 132 PTTS patients, an average of 1.5 interventions per patient, and 68.2% of patients required only one session. Interestingly, however, the final outcome was not associated with the number of interventions required (*p* = 0.162). This suggests that, if the patient's condition is suitable, there is no need to recoil from repeated interventions, as success and decannulation can be achieved even after multiple interventions. However, the cause of recurrent granulation tissue and the associated need for multiple interventions remains unclear. Moreover, apart from the total number of interventions per patient, neither the tracheostomy technique (PDT vs. surgical tracheostomy, *p* = 0.827) nor the number of tracheotomies (primary vs. re-tracheotomy, *p* = 0.621) nor presence of a tracheal cartilage fracture (*p* = 0.277) and the type of first intervention (*p* = 0.833) had a significant influence on the final outcome. However, for some parameters, only incomplete data was available (e.g., data on tracheal cartilage fractures was missing in 37.9% of patients due to referral from other hospitals) or subgroups were relatively small (re-tracheotomy: *n* = 8, 6.1%; surgical tracheotomy: *n* = 13, 9.9%; several types of initial interventions: *n* = 1–4, 0.8%–3.0%). Nevertheless, every patient may benefit from appropriate treatment of PTTS. In addition, there was no significant relation in the number of total interventions per patient regarding the tracheostomy technique (PDT vs. surgical tracheostomy, *p* = 0.891), number of tracheostomies (primary vs. re-tracheostomy, *p* = 0.640), presence of tracheal cartilage fracture (*p* = 0.277), type of initial intervention (*p* = 0.833), and final outcome (*p* = 0.162).

In this cohort, cryoablation was by far the most frequently performed procedure (117/198; 59.1%). In general, there is no consensus regarding the preferred method for interventional treatment of PTTS. Various modalities have been described: e.g., mechanical or balloon dilatation, stents or T-tubes, electrosurgery, lasers, APC, local drug administration (e.g., corticosteroids) and cryoablation (13, 21, 28, 29). Each of these methods has certain advantages and disadvantages (16). However, cryoablation allows an effective and tissue-sparing treatment with lower risk of serious complications such as perforation or bleeding (28, 30–32). Furthermore, cryoablation has been proven to be an effective and safe treatment for patients with PTTS (16). Nevertheless, other interventional methods must be available or complementarily used for unsuitable stenosis morphologies. When choosing the appropriate endoscopic modality there was no significant relation between the choice of the interventional modality and outcome or total number of interventions.

Furthermore, 70.5% of patients were treated with prednisolone and as many as 21.2% of patients were treated with prednisolone alone. This suggests that prednisolone appears to be sufficient as an anti-inflammatory and decongestant therapy in some patients. However, the majority still requires interventional treatment. This is consistent with the current literature (33, 34). We often use prednisolone as an adjunctive treatment for mucosal swelling, either as a pre-treatment to reduce the swelling before an intervention, and only sometimes as a definitive therapy.

Moreover, surgery and stents were only necessary in 2.3% and 0.8% of cases, respectively. This is advantageous because in previous studies on the effectiveness of various interventional modalities to treat benign tracheal stenosis (mostly PITS and/or PTTS) most complications arose either from stenting or surgical treatment (6, 11, 13, 15, 19–27).

Last but not least, the sex distribution was balanced (50.8% female) in the present study. However, it was interesting to note that tracheal cartilage fractures were statistically significantly more common in women (60.0% vs. 21.0%, *p* < 0.001). Because tracheal cartilage fractures predispose for tracheal stenosis (35), this is consistent with own unpublished data, which show that PTTS requiring treatment is significantly more likely to occur in women (OR: 2.8). However, a previous meta-analysis and a post-mortem study do not confirm these findings (36, 37). Furthermore, since most patients in our registry were referrals (74.2%), some data could not be collected in full, e.g., tracheal cartilage fractures (missing data in 37.9%). It is likely that the true incidence of tracheal cartilage fractures is significantly higher than reported here (22.0%). For this reason, tracheal cartilage fracture cannot be tested as a risk factor in the present study with sufficient statistical quality. However, own unpublished data show that the probability of PTTS requiring treatment is significantly higher (OR: 3.6) when tracheal cartilage fractures are present, which is in line with previous research (35). Since all patients were examined only endoscopically and not by cross-sectional imaging (computed tomography or magnetic resonance imaging) or ultrasound in clinical routine, intramucosal tracheal cartilage fractures may also have been missed contributing to underdiagnosis of tracheal cartilage fractures after tracheostomy. Another advantage of cross-sectional imaging could be a more accurate and therefore better grading of tracheal stenoses. However, endoscopic methods are more suitable for more dynamic stenoses. Therefore, complementary use appears to be most appropriate.

Despite being the largest (*n* = 132) of the few studies (*n* = 5–132) demonstrating safety and effectiveness of interventional treatment of PTTS the present study has some limitations (6, 11, 13, 15, 16, 19–22, 25–27). The external validity and thus transferability to the general population is limited by the study design (single-center, partly retrospective, potential selection bias). As only adults were included, the results cannot be transferred to pediatric patients. Additionally, different types of PTTS depending on stenosis site and pathophysiology have been described: subglottic, stoma, cuff, and tip type (38, 39). The subglottic type is usually caused by traumatic tracheostomy complications and the stomal type by bacterial infections and/or chondritis. The cuff type is considered to be caused by high cuff pressure and ischemic mucosal injury and the tip type is usually caused by excessive irritation by the stomal tip (38, 39). Although there seems to be no significant differences in baseline characteristics between PTTS types, patients with subglottic or stoma type stenosis may have better outcomes (39). In the present study all PTTSs were located directly above the tracheostoma and are therefore all subglottic PTTS. Therefore, outcomes for other types and differences in interventional success between different types could not be reported. Furthermore, since we only followed up for about a mean of 68 days, the long-term effectiveness of the interventional therapy in this study remains largely unclear. Moreover, the present study was not designed to compare different endoscopic modalities and their effectiveness. However, the direct comparison of the effectiveness of different interventional modalities is difficult, since their indications differ.

In conclusion, interventional treatment of post tracheostomy tracheal stenosis in neurological rehabilitation appears to be effective in enabling decannulation, and safe. Most patients only need one intervention. The need for stent implantation or surgical treatment is rare. Sometimes corticosteroids alone are sufficient. These results need to be verified in long-term, prospective, multi-center registries.

## Data Availability

The raw data supporting the conclusions of this article will be made available by the authors, upon reasonable request.
