# Supplementary material for: Interventional treatment of post tracheostomy tracheal stenosis in neurological rehabilitation: results of a single-center registry
Source: Front Rehabil Sci. 2026 Mar 12;7:1776925. doi: 10.3389/fresc.2026.1776925 (PMC13017792; doi:10.3389/fresc.2026.1776925)
Supplement: Supplementary file 1 [file Supplementaryfile1.docx]

Supplementary Material

**Supplementary Table 1.** Main and secondary diagnoses of the study cohort

| Main diagnosis | Secondary diagnoses | n (%) |
| --- | --- | --- |
| Critical illness polyneuropathy/myopathy | - History of cardiac surgery (Coronary artery bypass surgery, surgical aortic/mitral valve replacement, aortic dissection) - History of visceral surgery (intestinal ischemia, oesophageal rupture, oncologic hemicolectomy, perforated diverticulitis, acute abdominal aortic rupture) - Pneumonic sepsis, acutely exacerbated COPD, ARDS | 55 (41.7) |
| Haemorrhagic stroke | - Intracerebral haemorrhage (n=19) - Subarachnoid haemorrhage (n=14) | 33 (25.0) |
| Ischemic stroke |  | 21 (15.9) |
| Traumatic brain injury |  | 7 (5.3) |
| Subdural haemorrhage |  | 5 (3.8) |
| Cardiac arrest |  | 5 (3.8) |
| Pontine myelinolysis |  | 2 (1.5) |
| Glioblastoma |  | 1 (0.8) |
| Status epilepticus |  | 1 (0.8) |
| Meningitis |  | 1 (0.8) |
| Suicide attempt |  | 1 (0.8) |

**Supplementary Table 2.** Number of interventions stratified for tracheostomy technique

|  | Number of interventions | | | | | |
| --- | --- | --- | --- | --- | --- | --- |
|  | 1 (n=90) | 2 (n=25) | 3 (n=11) | 4 (n=5) | 5 (n=1) | Mean |
| Percutaneous dilatational tracheostomy (n=119), n (%) | 82 (68.9) | 22 (18.5) | 9 (7.6) | 5 (4.2) | 1 (0.8) | 1.50 |
| Surgical tracheostomy (n=13), n (%) | 8 (61.5) | 3 (23.1) | 2 (15.4) | 0 (0) | 0 (0) | 1.54 |

**Supplementary Table 3.** Number of interventions stratified for number of tracheostomies

|  | Number of interventions | | | | | |
| --- | --- | --- | --- | --- | --- | --- |
|  | 1 (n=90) | 2 (n=25) | 3 (n=11) | 4 (n=5) | 5 (n=1) | Mean |
| First Tracheostomy (n=124), n (%) | 86 (69.4) | 23 (18.6) | 10 (8.1) | 4 (3.2) | 1 (0.8) | 1.48 |
| Re-Tracheostomy (n=8), n (%) | 4 (50.0) | 2 (25.0) | 1 (12.5) | 1 (12.5) | 0 (0) | 1.88 |

**Supplementary Table 4.** Number of interventions stratified for presence of tracheal cartilage fracture

|  | Number of interventions | | | | | |
| --- | --- | --- | --- | --- | --- | --- |
|  | 1 (n=90) | 2 (n=25) | 3 (n=11) | 4 (n=5) | 5 (n=1) | Mean |
| Yes (n=29), n (%) | 17 (58.6) | 10 (34.5) | 1 (3.5) | 1 (3.5) | 0 (0) | 1.52 |
| No (n=53), n (%) | 37 (69.8) | 6 (11.3) | 6 (11.3) | 3 (5.7) | 1 (1.9) | 1.59 |
| Unknown (n=50), n (%) | 36 (72.0) | 9 (18.0) | 4 (8.0) | 1 (2.0) | 0 (0) | 1.40 |

**Supplementary Table 5.** Number of interventions stratified for modality of first intervention

|  | Number of interventions | | | | | |
| --- | --- | --- | --- | --- | --- | --- |
|  | 1 (n=90) | 2 (n=25) | 3 (n=11) | 4 (n=5) | 5 (n=1) | Mean |
| Prednisolone (n=30), n (%) | 23 (76.7) | 6 (20.0) | 1 (3.3) | 0 (0) | 0 (0) | 1.27 |
| Cryoablation (n=82), n (%) | 52 (63.4) | 18 (22.0) | 7 (8.5) | 4 (4.9) | 1 (1.2) | 1.59 |
| Argon plasma coagulation (n=2), n (%) | 1 (50.0) | 1 (50.0) | 0 (0) | 0 (0) | 0 (0) | 1.50 |
| Sling (n=12), n (%) | 10 (83.3) | 0 (0) | 2 (16.7) | 0 (0) | 0 (0) | 1.33 |
| External therapy (n=4), n (%) | 2 (50.0) | 0 (0) | 1 (25.0) | 1 (25.0) | 0 (0) | 2.25 |
| Surgery (n=1), n (%) | 1 (100) | 0 (0) | 0 (0) | 0 (0) | 0 (0) | 1.00 |
| Forceps (n=1), n (%) | 1 (100) | 0 (0) | 0 (0) | 0 (0) | 0 (0) | 1.00 |

**Supplementary Table 6.** Number of interventions stratified for outcome

|  | Number of interventions | | | | | |
| --- | --- | --- | --- | --- | --- | --- |
|  | 1 (n=90) | 2 (n=25) | 3 (n=11) | 4 (n=5) | 5 (n=1) | Mean |
| Successful decannulation (n= 102), n (%) | 74 (72.6) | 17 (16.7) | 7 (6.9) | 4 (3.9) | 0 (0) | 1.4 |
| Discharge with temporary tracheal cannula (n=1), n (%) | 0 (0) | 1 (100) | 0 (0) | 0 (0) | 0 (0) | 2.0 |
| Discharge with permanent tracheal cannula (n=23), n (%) | 13 (56.5) | 4 (17.4) | 4 (17.4) | 1 (4.4) | 1 (4.4) | 1.8 |
| Death (n=6), n (%) | 3 (50) | 3 (50) | 0 (0) | 0 (0) | 0 (0) | 1.5 |

**Supplementary Table 7.** Outcome stratified for tracheostomy technique

|  | Percutaneous dilatational tracheostomy (n=119), n (%) | Surgical tracheostomy (n=13), n (%) |
| --- | --- | --- |
| Successful decannulation (n= 102) | 95 (79.8) | 7 (53.9) |
| Discharge with temporary tracheal cannula (n=1) | 1 (0.8) | 0 (0) |
| Discharge with permanent tracheal cannula (n=23) | 18 (15.1) | 5 (38.5) |
| Death (n=6) | 5 (4.2) | 1 (7.7) |

**Supplementary Table 8.** Outcome stratified for number of tracheostomies

|  | First Tracheostomy (n=124), n (%) | Re-Tracheostomy (n=8), n (%) |
| --- | --- | --- |
| Successful decannulation (n= 102) | 97 (78.2) | 5 (62.5) |
| Discharge with temporary tracheal cannula (n=1) | 1 (0.8) | 0 (0) |
| Discharge with permanent tracheal cannula (n=23) | 21 (16.9) | 2 (25.0) |
| Death (n=6) | 5 (4.0) | 1 (12.5) |

**Supplementary Table 9.** Outcome stratified for presence of tracheal cartilage fracture

|  | Yes (n=29), (%) | No (n=53), n (%) | Unknown (n=50), n (%) |
| --- | --- | --- | --- |
| Successful decannulation (n= 102) | 23 (79.3) | 34 (64.2) | 45 (90.0) |
| Discharge with temporary tracheal cannula (n=1) | 0 (0) | 0 (0) | 1 (2.0) |
| Discharge with permanent tracheal cannula (n=23) | 5 (17.2) | 15 (28.3) | 3 (6.0) |
| Death (n=6) | 1 (3.5) | 4 (7.6) | 1 (2.0) |

**Supplementary Table 10.** Outcome stratified for number of interventions

|  | Number of interventions | | | | |
| --- | --- | --- | --- | --- | --- |
|  | 1 (n=90), n (%) | 2 (n=25), n (%) | 3 (n=11), n (%) | 4 (n=5), n (%) | 5 (n=1), n (%) |
| Successful decannulation (n= 102) | 74 (82.2) | 17 (68.0) | 7 (64.6) | 4 (80.0) | 0 (0) |
| Discharge with temporary tracheal cannula (n=1) | 0 (0) | 1 (4.0) | 0 (0) | 0 (0) | 0 (0) |
| Discharge with permanent tracheal cannula (n=23) | 13 (14.4) | 4 (16.0) | 4 (36.4) | 1 (20.0) | 1 (100) |
| Death (n=6) | 3 (3.3) | 3 (12.0) | 0 (0) | 0 (0) | 0 (0) |

**Supplementary Table 11.** Outcome stratified for modality of initial intervention

| Treatment | Outcome | | | |
| --- | --- | --- | --- | --- |
|  | Successful decannulation (n=102) | Discharge with temporary tracheal cannula (n=1) | Discharge with permanent tracheal cannula (n=23) | Death (n=6) |
| Prednisolone (n=30), n (%) | 23 (76.7) | 0 (0) | 5 (16.7) | 2 (6.7) |
| Cryoablation (n=82), n (%) | 63 (76.8) | 1 (1.2) | 14 (17.1) | 4 (4.9) |
| APC (n=2), n (%) | 0 (0) | 0 (0) | 2 (100) | 0 (0) |
| Sling (n=12), n (%) | 12 (100) | 0 (0) | 0 (0) | 0 (0) |
| External therapy (n=4), n (%) | 3 (75.0) | 0 (0) | 1 (25.0) | 0 (0) |
| Surgery (n=1), n (%) | 0 (0) | 0 (0) | 1 (100) | 0 (0) |
| Forceps (n=1), n (%) | 1 (100) | 0 (0) | 0 (0) | 0 (0) |

**Supplementary Figure 1.** Treatment process


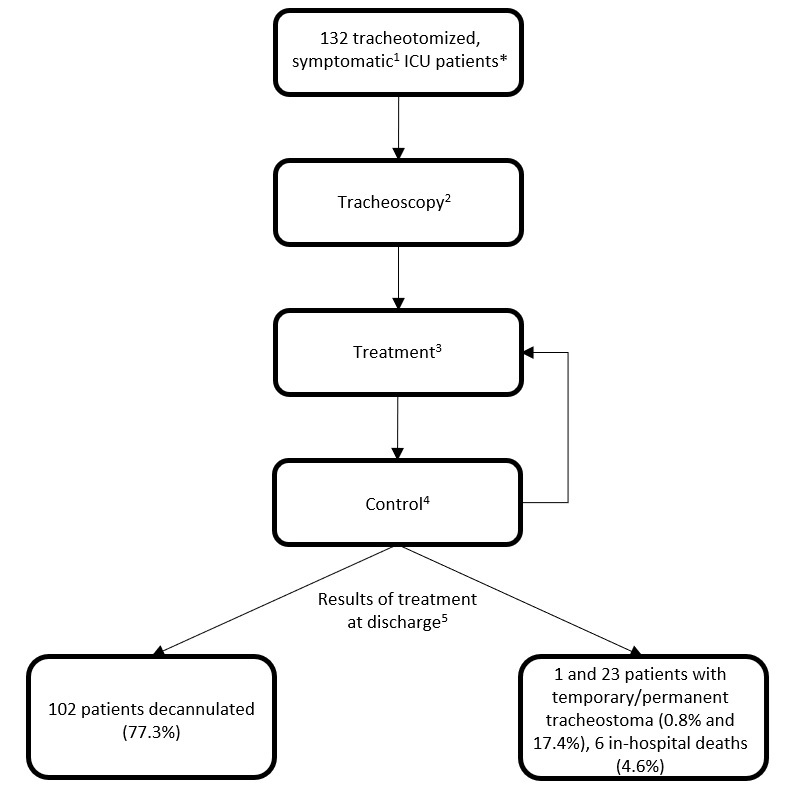


Legend: *: clinical evaluation before potential decannulation included unblocking of the tracheal cannula, visit by occupational therapist, linguist and FEES if necessary to exclude other potential causes for decannulation failure; 1: Exemplary symptoms leading to tracheoscopy: dyspnea, severe respiratory effort, stridor or drop in oxygen saturation; 2: see section 2.3; 3: see section 2.4 and Figure 1, treatment sometimes had to be repeated; 4: see section 2.3, seven to ten days after previous intervention; 5: mean follow-up duration of 61 ± 34 days
